# A Relative Deficiency of Lysosomal Acid Lypase Activity Characterizes Non-Alcoholic Fatty Liver Disease

**DOI:** 10.3390/ijms18061134

**Published:** 2017-05-25

**Authors:** Francesco Tovoli, Lucia Napoli, Giulia Negrini, Sergio D’Addato, Giulia Tozzi, Jessica D’Amico, Fabio Piscaglia, Luigi Bolondi

**Affiliations:** 1 Unit of Internal Medicine, Departmentt of Medical and Surgical Sciences, University of Bologna, 40136 Bologna, Italy; lucianapoli88@gmail.com (L.N.); giu.negrini@gmail.com (G.N.); sergio.daddato@unibo.it (S.D.); fabio.piscaglia@unibo.it (F.P.); luigi.bolondi@unibo.it (L.B.); 2 Unit of Neuromuscolar and Neurodegenerative Diseases, Children’s Hospital and Research Institute “Bambino Gesù”, 00165 Rome, Italy; giulia.tozzi@opbg.net (G.T.); jessica.damico@opbg.net (J.D.)

**Keywords:** non-alcoholic fatty liver disease, steatosis, steatohepatitis, lysosomal acid lipase, liver cirrhosis

## Abstract

Lysosomal acid lipase (LAL) is a key enzyme in lipid metabolism. Initial reports have suggested a role for a relative acquired LAL deficiency in non-alcoholic fatty liver disease (NAFLD)—however, it is still unclear whether this mechanism is specific for NAFLD. We aimed to determine LAL activity in a cohort of NAFLD subjects and in a control group of hepatitis C virus (HCV)-infected patients, investigating the role of liver cirrhosis. A total of 81 patients with a diagnosis of NAFLD, and 78 matched controls with HCV-related liver disease were enrolled. For each patient, LAL activity was determined on peripheral dried blood spots (DBS) and correlated with clinical and laboratory data. A subgroup analysis among cirrhotic patients was also performed. LAL activity is significantly reduced in NAFLD, compared to that in HCV patients. This finding is particularly evident in the pre-cirrhotic stage of disease. LAL activity is also correlated with platelet and white blood cell count, suggesting an analytic interference of portal-hypertension-induced pancytopenia on DBS-determined LAL activity. NAFLD is characterized by a specific deficit in LAL activity, suggesting a pathogenetic role of LAL. We propose that future studies on this topic should rely on tissue specific analyses, as peripheral blood tests are also influenced by confounding factors.

## 1. Introduction

Lysosomal acid lipase (LAL) is an enzyme that plays a pivotal role in lipid homeostasis, both in animal models and humans. Briefly, LAL hydrolyses cholesteryl esters and triglycerides internalized via receptor-mediated endocytosis of plasma lipoprotein particles (chylomicron remnants, intermediate density lipoproteins, and low-density lipoproteins) [1].

The resulting fatty acids and free cholesterol are key mediators in the homeostasis of the intra-cellular cholesterol pool [1].

In humans, genetically determined LAL deficiency is responsible for two distinct clinical entities: Wolman disease (WD) and cholesteryl ester storage disease (CESD) [2].

WD is a severe and life-threatening paediatric condition that usually leads to death within the first year of life, due to early-onset and rapidly evolving hepatic failure. On the other hand, CESD is a slowly evolving condition seen among adults, characterized by a heterogeneous and polymorphic clinical pattern. Manifestations of CESD include hepatosplenomegaly, micro/macrovesicular liver steatosis, early onset cardiovascular events, and dyslipidaemia [2].

The ability to diagnose these conditions has recently improved with the development of dried blood spot (DBS) testing. This test is able to evaluate the activity of LAL from a small sample of peripheral blood, allowing for a quick and minimally invasive diagnosis [3].

Moreover, sebelipase-alfa, a recombinant form of LAL, has been shown to be effective in the treatment of CESD, offering a therapeutic chance for these patients for the first time [4,5].

The widespread accessibility of reliable diagnostic tests, and the availability of a specific therapy, have inevitably raised the question of whether LAL activity is also altered in more common forms of liver steatosis, namely non-alcoholic fatty liver disease (NAFLD).

As such, hepatologists are showing increasing interest in LAL—in 2015, Baratta et al. [6] demonstrated that 240 consecutive patients with ultrasonographic evidence of NAFLD had reduced LAL activity, compared to 100 healthy subjects.

More interestingly, amongst the NAFLD population, a subgroup of patients with histology-proven non-alcoholic steatohepatitis (NASH) showed even more compromised LAL activity. The authors therefore suggested that impairment of LAL function could be associated with more aggressive NAFLD phenotypes [6].

A small study of 22 patients with liver steatosis demonstrated that lower levels of LAL activity were correlated with more advanced liver disease [7].

However, it is unclear whether LAL deficiency is a specific characteristic of NAFLD, or is shared with other different forms of chronic liver disease, such as viral hepatitis.

These doubts have been reinforced by a recent study by Vespasiani et al. [8], which did not find significant differences in LAL activity between patients with cryptogenic cirrhosis (which is often the result of chronic NASH), and patients with liver cirrhosis secondary to other aetiologies. Both groups, however, showed impaired LAL activity in comparison to healthy controls.

Furthermore, the same described an analytic interference by platelet count on LAL-levels [8] thus instilling doubts on the previously described correlation between LAL activity and NAFLD severity.

Consequently, a consensus regarding the role of LAL deficiency in chronic liver disease has yet to be established.

As the Baratta and Vespasiani studies involved only patients with early and advanced liver disease, respectively, current uncertainties may arise from the lack of studies evaluating patients with both early and advanced liver disease.

The aim of our study was to analyse LAL activity in a population of cirrhotic and non-cirrhotic patients diagnosed with NAFLD, compared to that in a matched population of patients with HCV-related chronic liver disease.

We also aimed to investigate the role of liver cirrhosis and identify other epidemiological, clinical, and biochemical parameters associated with LAL activity.

## 2. Results

### 2.1. Study Population

The study population included 81 NAFLD and 78 HCV patients.

As shown in Table 1, the two groups had similar demographic characteristics in terms of age and sex. Prevalence of liver cirrhosis was also similar in the NAFLD and HCV groups.

No significant differences were found in terms of ALT, AST, albumin, and total bilirubin. Both the Child-Pugh and MELD scores were similar, and haematological tests were also comparable.

A statistically significant but clinically-irrelevant difference in INR was present.

As expected, the total cholesterol, low density lipoprotein (LDL) cholesterol, and triglycerides values were significantly higher in the NAFLD group.

Additionally, the prevalence of diabetes mellitus was significantly higher in the NAFLD group, as well as the rate of patients treated with metformin and statins.

Other drugs taken by the patients at the enrolment included: potassium canrenoate (15 patients), furosemide (9 patients), β-blockers (8 patients), proton pump inhibitors (8 patients), angiotensin-converting enzyme inhibitors (6 patients), angiotensin II receptor blockers (3 patients), glicazide (2 patients), and pioglitazone (1 patient). The rate of patients treated with these drugs did not differ between the two groups.

### 2.2. LAL Activity in NAFLD and in HCV Patients–Whole Study Population

**Figure 1:**
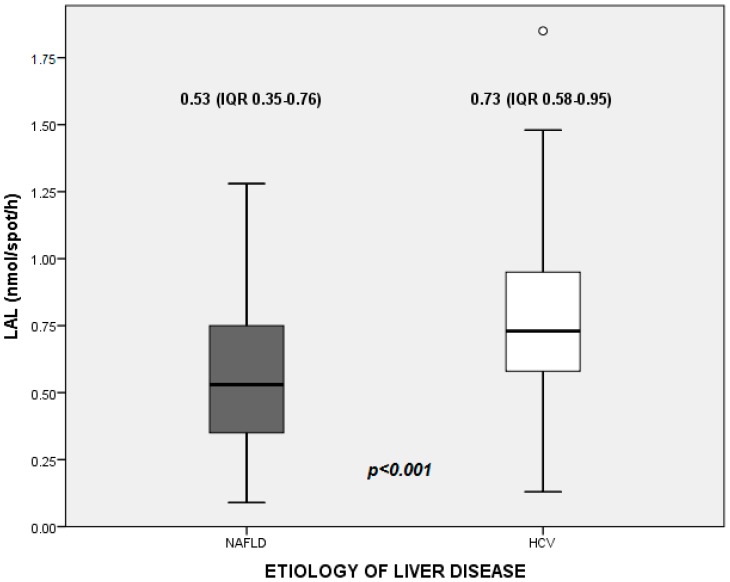
Activity of lysosomal acid lipase (LAL) as measured on peripheral blood of patients with with non-alcoholic fatty liver disease (NAFLD) and hepatitis C virus (HCV)-related chronic liver disease.

The median value of LAL activity in the whole study population was 0.66 nmol/spot/h (0.46–0.86). In detail, median values were 0.53 (0.35–0.76) nmol/spot/h in NAFLD patients, and 0.73 (0.58–0.95) nmol/spot/h in HCV patients (Figure 1).

For the univariate analyses, both NAFLD (*p* < 0.001) and liver cirrhosis (*p* = 0.005) were associated with LAL-activity below median (Table 2).

Furthermore, oesophageal varices (*p* = 0.021) were also related to lower levels of LAL. Additionally, we found an inverse correlation with spleen sectional area (*p* = 0.006), and a direct correlation with total white blood cell count (*p* = 0.002), neutrophils (*p* = 0.005), monocytes (*p* = 0.021), and platelets levels (*p* < 0.001).

Other factors related to LAL activity at the univariate analyses included diabetes mellitus (*p* = 0.046), INR (*p* = 0.042), and MELD (*p* = 0.037). A marginal correlation was also noted for HCC (*p* = 0.051), lymphocyte count (*p* = 0.082), HDL cholesterol (*p* = 0.084), statin (*p* = 0.059), and metformin treatments (*p* = 0.050).

All of the aforementioned parameters were included in the multivariate model, with the exception of total white blood cell count due to co-linearity.

Following backward analyses, the only factors significantly associated with LAL activity below the median were NAFLD (Exp (B) = 4.511, CI 2.138–9.519, *p* < 0.001), platelet count (Exp (B) = 1.009, CI 1.004–1.015, *p* = 0.001), and neutrophil count (Exp (B) = 1.001, CI 1.000–1.001, *p* = 0.048).

### 2.3. Subgroup Analyses: LAL Activity in Cirrhotic Patients

NAFLD and HCV patients shared similar demographic and laboratory characteristics also in the subgroups (Table 3).

The median LAL activity in cirrhotic patients was 0.59 (0.41–0.79) nmol/spot/h, with a statistically significant difference between NAFLD and HCV patients (0.53 (0.29–0.69) vs. 0.67 (0.50–0.89) nmol/spot/h, Mann–Whitney *u* = 654.5, *p* = 0.005).

Variables that were either fully or marginally correlating with LAL activity below median at the univariate analyses included: age (*p* = 0.028), NAFLD (*p* = 0.046), HCC (*p* = 0.073), total white blood cell count (*p* = 0.007), neutrophils(*p* = 0.064), lymphocytes (*p* = 0.005), monocytes (*p* = 0.056) and platelet levels (*p* = 0.001), INR (*p* = 0.008), metformin treatment (*p* = 0.082), and spleen sectional area (*p* = 0.005) (Table 4).

White blood cell count and metformin treatment were excluded from the multivariate model due to co-linearity (the latter strongly correlating with NAFLD).

The binary logistic regression, performed according to the same methods used in the evaluation of the whole study population, confirmed a statistically significant association between values of LAL activity below median and NAFLD (Exp (B) = 3.325, CI 1.185–9.365, *p* = 0.023) and platelets (Exp (B) = 1.011, CI 1.001–1.021, *p* = 0.028).

### 2.4. Subgroup Analyses: LAL Activity in Non-Cirrhotic Patients

Demographic and laboratory characteristics of NAFLD and HCV non-cirrhotic patients were comparable, with the exception of a clinically-irrelevant difference in INR (Table 3). As found in the whole study population, the rate of patients treated with statins, as well as cholesterol and tryglicerides levels, were higher in the NAFLD group.

The median value of LAL activity in this cohort was 0.74 (0.48–0.97) nmol/spot/h, with a statistically significant difference found between NAFLD and HCV patients (0.55 (0.41–0.81) vs 0.84 (0.69–1.07) nmol/spot/h, Mann–Whitney *u* = 304, *p* < 0.001).

Univariate analyses showed that NAFLD (*p* = 0.005), white blood cells (*p* = 0.037), neutrophils (*p* = 0.048), and monocyte count (*p* = 0.081) were the only other factors correlated with LAL activity (Table 5).

White blood cells were excluded from the multivariate analyses due to co-linearity.

The binary logistic regression confirmed a statistically significant association between values of LAL activity below median and NAFLD (Exp (B) = 5.135, CI 1.752–15.053, *p* = 0.003) and neutrophils (Exp (B) = 1.000, CI 1.000–1.001, *p* = 0.026), but no other variables.

### 2.5. Stratification According to Platelet and White Blood Cells Count

To further clarify the association between NAFLD and reduced LAL-activity avoiding the interferences from platelet and white blood cell count, we performed further analysis stratifying the whole study population according to these parameters.

First, we divided the study population according to the tertiles of platelet count, irrespective of the etiology of liver disease. The following groups were created—Group 1: platelet count (38–122) × 10^3^/mmc (53 patients); Group 2: platelet count (123–201) × 10^3^/mmc (53 patients); and Group 3: platelet count (202–412) × 10^3^/mmc (53 patients).

Then, we proceeded with the subgroup analyses in the same methodological line already adopted in the analyses of whole study population. Median values of LAL-activity were as follows: 0.52 nmol/spot/h (0.34–0.69) in Group 1; 0.69 nmol/spot/h (0.50–0.84) in Group 2; and 0.80 nmol/spot/h (0.56–0.98) in Group 3, respectively. NAFLD etiology was confirmed as an independent predictor of LAL-activity below median in all of the subgroups (Group 1: Exp (B) 3.200, CI 1.039–9.852, *p* = 0.043; Group 2: Exp (B) 3.239, CI 1.041–10.074, *p* = 0.042; Group 3: Exp (B) 7.755, CI 2.286–26.305, *p* = 0.001).

Using the same procedure, a final analysis was performed dividing the whole study population according to the tertiles of white blood cells count. The following groups were created—Group 1: white blood cells count 1470–4530/mmc (53 patients); Group 2: white blood cells count 4531–6614/mmc (53 patients); and Group 3: white blood cells count 6615–11380/mmc (53 patients). Median values of LAL-activity were 0.53 nmol/spot/h (0.32–0.76) in Group 1; 0.68 nmol/spot/h (0.43–0.96) in Group 2; and 0.74 nmol/spot/h (0.59–0.95) in Group 3, respectively. Even in this analysis, NAFLD was an independent predictor of LAL-activity below median in all of the subgroups (Group 1: Exp (B) 4.343, CI 1.344–14.030, *p* = 0.014; Group 2: Exp (B) 4.886, CI 1.530–15.605, *p* = 0.007; Group 3: Exp (B) 3.800, CI 1.212–11.918, *p* = 0.022).

## 3. Discussion

NAFLD is a condition characterized by pathologic intrahepatic lipid accumulation in the absence of significant alcohol intake and secondary causes [9,10]. There is growing interest in this condition as its more aggressive form (i.e., NASH) is becoming a leading cause of liver cirrhosis and HCC [11]. Although insulin resistance is a key factor in the development of NAFLD [9], other pathogenic factors are also involved—identification of these elements may contribute to a better understanding of this condition.

LAL, a key enzyme in regulating intrahepatic lipid trafficking, may represent one of these factors. Furthermore, previous reports have suggested that LAL activity is reduced in NAFLD patients, compared to that in healthy subjects.

We evaluated LAL activity in a large population, which included both NAFLD and HCV patients; for the first time, subgroup analyses were also performed for cirrhotic and non-cirrhotic patients.

Two main conclusions can be drawn from our study. First, NAFLD was independently associated with lower values of LAL activity, compared to HCV-related chronic liver disease. This finding is particularly evident in the whole study population and in non-cirrhotic patients. In contrast, there is a weaker effect with a borderline statistical significance in the subgroup of cirrhotic patients.

These are novel findings, and shed a new light on the pathogenesis of NAFLD, as we demonstrated for the first time that a relative deficiency in LAL activity is specific for NAFLD and appears since the early stages of the disease.

Some limitations of our study have to be considered. A first main limitation is represented by the absence of a systematic histological confirmation of NAFLD. Liver biopsy is associated with known risks, and its systematic use outside clinical trials, such as in the case of our study, may pose ethical problems. Furthermore, current guidelines do not support the systematic use of liver biopsy, preferring a case-by-case selection based on non-invasive markers of fibrosis [9,10]. The design of our study reflects these recommendations, and is similar to the design of other studies examining LAL activity in human adults [6,8]. Furthermore, it must be stressed that misdiagnosis and/or misclassification of NAFLD subjects is extremely unlikely, as ultrasonographic diagnosis of liver steatosis has a specificity as high as 95–99% [12,13,14]. Finally, in our study, the ultrasonographic diagnosis of liver steatosis was confirmed by expert operators performing real-time imaging. In this setting, the inter-rater variability is limited and comparable with that of biopsy data, as shown in a large meta-analysis [15,16].

The second main limitation of the study resides in its cross-section design, similar to all of previous studies on this topic. Therefore, it is impossible to state whether the reduced LAL activity of NAFLD patients is the cause or an epiphenomenon of the dysmetabolic liver disease.

In any case, evidences derived from previous studies performed in patients with CESD show that a reduction in LAL activity contributes to the accumulation of intracellular cholesterol in liver cells, ultimately leading to cellular death and fibrosis [2,17]. Therefore, it could be speculated that the relative LAL deficiency occurring in NAFLD patients may contribute to the lipid-induced liver damage and its progression. Interestingly, it has been recently hypothesized that changes in LAL activity could contribute to the atherosclerotic process promoting the formation and the accumulation of foam cells within wall arteries [17]. Therefore, it could be speculated that LAL deficiency may also be involved in the pathogenesis of the extra-hepatic manifestations of NAFLD. Obviously, this hypothesis will need confirmation in future studies.

To compare our data with those found in current literature, it was necessary to take into account the different populations involved in previous studies. Baratta et al. [6] evaluated a population mostly composed of non-cirrhotic patients. On the other hand, presence of liver cirrhosis was a required inclusion criterion in the study by Vespasiani et al. [8].

Taking into account these differences, our study (which included similar proportions of both cirrhotic and non-cirrhotic patients), integrated previous findings of Baratta et al. [6], demonstrating for the first time that a relative reduction in LAL activity differentiates NAFLD not only from healthy subjects, but also from pathological viral controls.

At first glance, our data appear to be conflicting with the previous study by Vespasiani et al. [8], which failed to demonstrate a significant difference of LAL activity in patients with cryptogenic cirrhosis, compared to other causes of liver cirrhosis such as viral hepatitis. In truth, our data are not in full conflict with these previous findings. In fact, within the subgroup analyses of cirrhotic patients, the multivariate model revealed only a weak statistical correlation between aetiology and LAL activity. The regression coefficient for NAFLD is lower in this subgroup compared to the whole population and to non-cirrhotic patients. Furthermore, the statistical significance is also weak in this subgroup. Therefore, the difference in LAL-activity in the subgroup of cirrhotic patients is particularly difficult to be demonstrated. Two different reasons may justify this finding. First, the peculiar features characterizing the active phase of the different etiologies of liver damage (i.e., viral, alcoholic, dismetabolic, autoimmune, etc.) progressively disappear in parallel to the cirrhotic scarring process. In the specific case of NAFLD, as fibrosis progresses, intra-hepatic lipid content reduces and steatosis tends to disappear. Therefore, it is likely that differences in LAL levels are more pronounced in the early stage of disease (when steatosis is still well-represented) and less evident in the late stages (due to the confounding effect of fibrosis). Second, in a setting of lower absolute values of LAL-activity, even slight pre-analytic or analytic confounding factors may lead to different statistical results.

As a second major finding, our study revealed that LAL activity detected on peripheral blood samples is also significantly affected by circulating platelets. This finding is not surprising, as LAL is an enzyme potentially expressed by any cellular lines containing lysosomes—both white blood cells [18] and platelets [19] possess lysosomal structures.

In the setting of a complete and genetically determined LAL deficiency (such as in the case of WD and CESD), the enzymatic pool detectable on peripheral blood samples reliably reflects the actual hepatic pool. This is the reason behind the reliable diagnostic accuracy of DBS tests for the genetic causes of NAFLD.

Instead, in patients with thrombocytopenia, the circulating pool can no longer be reliably representative of the hepatic pool.

This aspect, which validate similar findings described in Vespasiani et al.’s [8] study, is not a limit to our results as both liver disease aetiology and platelet count are independently correlated to LAL-activity. Moreover, since our NAFLD and HCV study populations were comparable in terms of platelet count, this analytic interference did not influence our results.

Instead, our study shed a new light on previous papers which assessed a correlation between LAL-activity and disease severity.

This link was initially suggested in the work by Baratta et al. [6], which showed a lower LAL-activity in patients with histology proven NASH compared to the other NAFLD subjects. Later, Shteyer et al. [7] showed that a low serum LAL-activity correlated with advanced liver disease. Finally, a recent paper by Selvakumar et al. [20] correlated LAL deficiency with the severity of liver fibrosis in 168 consecutive children with biopsy-proven NAFLD.

None of these studies, however, included a systematic evaluation of the platelet count of the enrolled patients.

Although these papers certainly were innovative in suggesting a possible role of LAL in NAFLD and brought useful insights, it is entirely possible that thrombocytopenia may have acted as a confounding factor in the more severe cases. To this end, we did not find a correlation between LAL-activity and disease severity at the multivariate analysis, as already described in Vespasiani’s study [8].

Based on our results, we suggest that findings from previous studies correlating LAL-activity with NAFLD severity without a systematic determination of platelet count should be considered with caution.

Further, due to the platelet count interference, comparison between LAL-activity in healthy controls and patients with different grades of chronic liver disease is hardly informative.

In view of this, future studies regarding the role of LAL in the pathogenesis of NAFLD should focus on tissues, as opposed to peripheral blood.

To the best of our knowledge, there are no data in the literature evaluating the tissutal expression of LAL in liver samples of NAFLD patients. Simultaneous evaluation of LAL in peripheral blood and liver samples of NAFLD patients undergoing confirmatory biopsy (or liver resection for HCC) may be a promising target for future studies. With this background, the hypothesis of therapeutic implication of sebelipase alfa is currently considered to be premature.

## 4. Materials and Methods

### 4.1. Study Population

Consecutive patients referred to the Internal Medicine Unit of the Bologna Authority Hospital Sant’Orsola-Malpighi, both as inpatients and as outpatients, were enrolled in this study.

The study group included patients diagnosed with NAFLD, while patients with HCV-related chronic liver disease were enrolled into the control group.

The study and control groups were matched for age, sex, and stage of liver disease (in particular, according to the presence or absence of established liver cirrhosis).

Enrolled patients underwent a venepuncture after a 12-h fast to obtain a small sample of peripheral blood (<1 cc). This sample was used to perform the diagnostic test evaluating LAL activity.

The LAL activity results were correlated with clinical and laboratory parameters, which included age, sex, body mass index, diagnosis of hepatocellular carcinoma (HCC), portal hypertension, hepatic encephalopathy and diabetes mellitus, as well as levels of neutrophils, lymphocytes, monocytes, platelets, international normalized ratio (INR), serum creatinine, albumin, alanine aminotransferase (ALT), aspartate aminotransferase (AST), total bilirubin, total cholesterol, triglycerides, Child-Pugh score, model for end-stage liver disease (MELD) score, and spleen sectional area. Treatment with statin was also evaluated, as it was shown to correlate with LAL activity in a prior study [6].

To allow corroboration with previous papers on this topic, two subgroup analyses in cirrhotic and non-cirrhotic cohorts, respectively, were also performed.

All participating patients signed an informed consent before the study. The study was approved by the ethics committee of the Bologna Authority Hospital Sant’Orsola-Malpighi (protocol 45/2016/U/Tess, 2016), and conducted according to the ethical guidelines of the 1975 Declaration of Helsinki.

### 4.2. Assessment of NAFLD

A diagnosis of NAFLD was established, according to the European Association for the Study of the Liver (EASL) guidelines [9]. Both evidence of liver steatosis and exclusion of differential diagnosis were required.

The main inclusion criterion was based on ultrasonographic evidence of liver steatosis, defined according to the Hamaguchi criteria (abnormally intense, high level echoes arising from the hepatic parenchyma, liver-kidney difference in echo amplitude, echo penetration into the deep portion of the liver and clarity of liver blood vessel structure) [12].

In all of the enrolled patients, ultrasonographic evidence of steatosis was confirmed by a member of our équipe, with a minimum expertise of 10 years in ultrasound imaging, and performing at least 1000 ultrasound examinations per year. Assessment of steatosis was performed with real-time imaging. On the contrary, assessment based on the examination of previously acquired static images was not allowed.

All of the participants were required to be at least 18 years old. Patients were also required to be able to express a valid informed consent, and to comply with the venepuncture required by the study protocol.

The exclusion criteria included prior or current alcohol intake of >20 g/day, positive tests for hepatitis B virus (HBV) and/or HCV infection, use of drugs known to induce chronic liver damage, and other known causes of chronic liver disease (e.g., autoimmune liver disorders, glycogenosis, and other storage disease). Additionally, the following laboratory findings were also considered exclusion criteria: antinuclear antibodies and/or anti-smooth muscle antibodies >1:80, positive anti-mitochondrial antibodies, positive anti kidney-liver microsomal antibodies, transferrin saturation >45%, ceruloplasmin serum levels <20 mg/dL, and α1-antitrypsin <90 mg/dL.

A systematic confirmation of NAFLD by the means of liver biopsies outside clinical trials (as in the case of our study), is not supported by the current guidelines [9,10], and would pose ethical concerns. As recommended, histology confirmation was obtained from the patients with non-invasive markers (Fibroscan, NAFLD fibrosis score, and Fibrosis4-score), suggestive of advanced liver disease (*n* = 46) [9].

### 4.3. Assessment of HCV-Related Chronic Liver Disease

All the patients included in this group demonstrated positive anti-HCV antibodies and detectable HCV-RNA at the time of enrolment.

Patients with multifactorial chronic liver disease (e.g., HCV-HBV co-infected patients, HCV patients with current or prior history of significant alcohol consumption) were excluded from the study. For the same reasons, HCV patients with ultrasonographic evidence of fatty liver were also excluded from the study. The latter choice was also performed to avoid the possible confounding aspects induced by steatogenic effect of HCV genotypes 2 and 3.

### 4.4. Assessment of Liver Cirrhosis

Cirrhosis was diagnosed on standard clinical, laboratory, ultrasound, elastosonographic, and/or histology examinations.

Since steatosis usually disappears in cirrhotic livers, the simultaneous ultrasonographic diagnosis of NAFLD and cirrhosis may be problematic. As such, for patients included in the “cirrhotic NAFLD” subgroup, the diagnosis of NAFLD always preceded the documentation of liver cirrhosis. In different words, the subgroup was composed by NAFLD patients who had developed cirrhosis during their clinical follow-up. On the contrary, patients with liver cirrhosis classified as NAFLD only on a clinical basis (i.e., cryptogenic cirrhosis in patients with features of metabolic syndrome) were not included in the study.

### 4.5. Lysosomal Acid Lipase (LAL) Activity Assay

A buffer solution was prepared with 0.15 M acetate buffer at pH 4.0 containing 1.0% Triton X-100. 1.0 mL 0.5% (*w*/*v*) cardiolipin (in methanol) and 400 μL (13.3 mM) 4MU-palmitate (in DMSO) were added to 14 mL buffer solution. 30 μM Lalistat-2 was prepared fresh each time by diluting 200 μM Lalistat2 (in DMSO) with distilled water. Cardiolipin (sodium salt, bovine), sodium acetate (trihydrate), 4-methylumbelliferone (4-MU), dimethyl sulfoxide (DMSO), mercuric chloride, and Triton X-100 were obtained from Sigma Aldrich Company Ltd. (Dorset, UK). 4-MU palmitate and Lalistat2 were supplied by Apollo Scientific (Bredbury, UK) and Alexion (Cheschire, CT, USA), respectively.

LAL activity in DBS was measured as reported by Hamilton et al. [3], using Lalistat2 as a specific inhibitor, and cardiolipin as an activator for LAL.

A blood sample collected into an ethylenediaminetetraacetic acid tube was spotted onto a filter paper (Whatman grade 903 Schleicher & Schuell), according to the requirements of the National Committee for Clinical Laboratory Standard (NCCLS) protocol. A total of 75 μL blood was spotted on DBS cards and allowed to dry overnight at room temperature. Samples were stored double-bagged with desiccant at −20 °C, and analysed within 1 week of collection. Alternatively, they may be stored at a temperature of −20 °C and analysed within 1 month. Uninhibited and inhibited activities with Lalistat 2 were performed. LAL activity was determined by subtracting activity in the inhibited reaction from uninhibited reaction (total lipase), and expressed as nmol/spot/h of 4MU (methylumbelliferone) [3]. The reaction was stopped with 100 µL stop buffer HgCl_2_ (15 mM). A 0–2.5 nmol/well 4MU standard was built. The fluorescence intensity was measured using the Enspire Multimode plate reader by Perkin Elmer (Waltham, MA, USA) (λ excitation = 355 nm, λ emission = 460 nm).

### 4.6. Sample Size Calculation

At the start of study, there were no data describing LAL activity in patients with liver cirrhosis, nor in patients with HCV infection. Therefore, the sample size had been calculated hypothesizing a LAL activity in HCV patients approximately halfway of that observed in NAFLD patients and healthy controls. The number of patients to include was calculated by a 2-tailed student t-test for independent variables, considering a standard deviation between the two groups SD = 0.35 (as previously described [6]), and a mean difference of 0.32 nmol/spot/h. With a type I error α = 0.05, the inclusion of 33 patients in each group guaranteed a power of 90%.

To allow a reliable comparison between subgroups, the enrolment was extended to approximately twice of the calculated sample size.

### 4.7. Statistical Analysis

Distributions of continuous variables were tested using the Kolmogorov-Smirnov test. Data are expressed as mean (standard deviation), or median (range), where appropriate.

Group comparisons were performed with unpaired Student’s *t*-tests for normally distributed variables, and the Mann–Whitney or Kruskall–Wallis test for non-normally distributed variables.

Categorical variables were tested using the 2-tailed Fisher’s test.

Distribution of LAL activity in each group was expressed as a median and interquartile range.

A binary logistic regression was performed using levels of LAL activity below the median as the dependent variable.

Variables, whose association with LAL activity at the univariate analysis showed a *p* < 0.10, were entered into the multivariate models, in order to identify factors independently associated with LAL function. A *p* < 0.05 was considered a cut-off for statistical significance.

Statistical analyses were performed with SPSS version 20.0 (SPSS Inc., Chicago, IL, USA) [5].

## 5. Conclusions

In conclusion, our findings demonstrated that NAFLD patients show a relative deficit of LAL activity from the early stages of disease, suggesting a possible pathogenic role of LAL in NAFLD, and paving the way for promising studies on this topic. The interference of platelet count on DBS-detected LAL activity, although not limiting our findings, suggest the opportunity of using tissues different from peripheral blood for future research regarding the role of LAL in chronic liver disease.

## Figures and Tables

**Table 1:** Main demographic, clinical, and laboratory findings of patients with non-alcoholic fatty liver disease (NAFLD) and hepatitis C virus (HCV)-related chronic liver disease. Continuous variables are expressed as median (range). Categorical variables are espressed as number of patients (percentage).

Variable	NAFLD (*N* = 81)	HCV (*N* = 78)	*p*
Age (years)	60 (18–85)	63 (35–86)	0.092
Sex (M/F)	53/28 (65%/35%)	41/37 (53%/47%)	0.109
Body mass index (kg/m^2^)	27.8 (22.2–40.2)	23.6 (19.4–29.8)	0.001
White blood cells (/mmc)	5820 (1950–10930)	5515 (1470–11380)	0.569
Neutrophils (/mmc)	3760 (1150–9240)	3130 (960–8800)	0.204
Lymphocytes (/mmc)	1500 (180–4500)	1385 (300–4530)	0.789
Platelets (10^3^/mmc)	171 (41–400)	143 (38–412)	0.964
INR (ratio)	1.05 (0.82–1.90)	1.11 (0.90–2.09)	0.012
Albumin (g/L)	39.0 (18.0–49.0)	37.8 (18.0–47.7)	0.548
Creatinin (mg/dL)	0.81 (0.38–3.00)	0.78 (0.38–1.52)	0.068
ALT (IU/L)	35 (10–188)	40.5 (10–345)	0.445
AST (IU/L)	32 (5–135)	35 (6–226)	0.407
Bilirubin (mg/dL)	0.82 (0.26–8.30)	0.87 (0.28–5.96)	0.919
Total cholesterol (mg/dL)	171 (69–327)	159 (47–239)	0.034
HDL cholesterol (mg/dL)	45 (9–97)	47.5 (8–132)	0.513
LDL cholesterol (mg/dL)	100.8 (17–231)	86.8 (25–163)	0.006
Tryglicerides (mg/dL)	90 (19–278)	70 (32–245)	0.001
Cirrhosis (*n*)	43 (53.1%)	46 (59.0%)	0.520
Spleen section area (cm^2^)	51 (21–180)	48 (22–150)	0.311
Ascites (%)	13 (16.0%)	9 (11.5%)	0.494
Diabetes (%)	32 (39.5%)	11 (14.1%)	<0.001
Metformin (%)	16 (19.8%)	2 (2.6%)	0.001
Insulin (%)	12 (14.8%)	5 (6.4%)	0.123
Statin (%)	18 (22.2%)	2 (2.6%)	<0.001

INR: international normalized ratio; AST: aspartate aminotranferase; ALT: alanine aminotranferase; HDL: high-density lipoprotein; LDL: low-density lipoprotein.

**Table 2:** Univariate and multivariate binary logistic regression analysis to lysosomal acid lipase (LAL) in the study population as a whole (performed using LAL below median as the dependent variable).

Univariate				Variable	Multivariate			
Exp (B)	95% CI for B		*p*		Exp (B)	95% CI for B		*p*
0.992	0.971	1.014	0.473	Age (years)				
0.837	0.445	1.577	0.583	Sex (F = 0, M = 1)				
3.387	1.766	6.495	<0.001	Etiology (NAFLD = 1, HCV = 0)	4.511	2.138	9.519	<0.001
2.491	1.309	4.742	0.005	Cirrhosis (NO = 0, YES = 1)	1.137	0.434	2.96	0.793
1.998	0.996	4.008	0.051	HCC (NO = 0, YES = 1)	0.864	0.358	2.086	0.745
2.320	1.126	4.736	0.021	Oesophageal varices (NO = 0, YES = 1)	0.797	0.279	2.265	0.625
2.054	0.773	5.458	0.149	Ascites (NO = 0, YES = 1)				
1.000	1.000	1.000	0.002	White blood cells (×10^3^/mm^3^)	*			
1.000	0.999	1.000	0.005	Neutrophils (×10^3^/mm^3^)	1.000	0.999	1.000	0.048
1.000	0.999	1.000	0.082	Lymphocytes (×10^3^/mm^3^)	1.000	0.999	1.000	0.761
0.998	0.997	1.000	0.021	Monocytes (×10^3^/mm^3^)	1.000	0.998	1.002	0.906
0.991	0.986	0.995	<0.001	Platelets (×10^3^/mm^3^)	0.991	0.986	0.996	0.001
10.350	1.093	98.04	0.042	INR (ratio)	15.873	0.989	250.001	0.061
0.954	0.549	1.657	0.866	Albumin (g/dL)				
1.072	0.449	2.559	0.875	Creatinin (mg/dL)				
0.995	0.986	1.003	0.226	ALT (IU/L)				
0.995	0.987	1.004	0.274	AST (IU/L)				
1.255	0.914	1.723	0.161	Total bilirubin (mg/dL)				
1.003	0.998	1.008	0.111	Body mass index (kg/m^2^)				
0.994	0.987	1.001	0.106	Total cholesterol (mg/dL)				
0.986	0.971	1.002	0.084	HDL cholesterol (mg(dL)	0.987	0.969	1.006	0.176
0.995	0.986	1.003	0.219	LDL cholesterol (mg/dL)				
1.000	0.994	1.007	0.939	Tryglycerides (mg/dL)				
2.077	1.012	2.641	0.046	Diabetes (NO = 0, YES = 1)	0.961	0.299	3.088	0.947
2.656	0.965	7.313	0.059	Statin (NO = 0, YES = 1)	2.588	0.769	8.716	0.125
2.955	0.115	1.000	0.050	Metformin (NO = 0, YES = 1)	2.095	0.558	2.569	0.254
1.157	0.442	3.169	0.776	Insulin (NO = 0, YES = 1)				
1.019	1.005	1.032	0.006	Spleen sectional area (cm^2^)	1.001	0.983	1.019	0.950
1.039	0.862	1.253	0.687	Child-Pugh score				
1.127	1.007	1.261	0.037	MELD Score	1.064	0.917	1.236	0.413

* Excluded for multivariate analyses due to co-linearity. INR: international normalized ratio; AST: aspartate aminotranferase; ALT: alanine aminotranferase; HDL: high-density lipoprotein; LDL: low-density lipoprotein.

**Table 3:** Main demographic, clinical, and laboratory findings of patients with non-alcoholic fatty liver disease (NAFLD) and hepatitis C virus (HCV)-related chronic liver disease, divided according to the presence (left column) or the absence (right column) of liver cirrhosis. Continuous variables are expressed as median (range). Categorical variables are espressed as number of patients (percentage).

Variable	NAFLD (*N* = 43)	HCV (*N* = 46)	*p*		NAFLD (*N* = 38)	HCV (*N* = 32)	*p*
Age (years)	64 (20–85)	66.5 (44–86)	0.303	Age (years)	55 (18–84)	58.5 (35–82)	0.227
Sex (M/F)	27/16	26/20	0.666	Sex (M/F)	26/12	15/17	0.090
White blood cells (/mmc)	5120 (1950–10000)	5005 (1470–11380)	0.790	White blood cells (/mmc)	6260 (3230–10930)	5880 (3220–9570)	0.892
Neutrophils (/mmc)	3440 (1150–6740)	2925 (960–8800)	0.343	Neutrophils (/mmc)	4010 (1770–9240)	3705 (1700–7670)	0.667
Lymphocytes (/mmc)	1230 (180–4500)	1305 (300–4530)	0.353	Lymphocytes (/mmc)	1820 (639–3510)	1565 (610–4530)	0.416
Monocytes (/mmc)	430 (50–1180)	400 (80–1310)	0.755	Monocytes (/mmc)	440 (30–1500)	475 (190–990)	0.608
Platelets (10^3^/mmc)	109 (41–286)	123.5 (38–363)	0.687	Platelets (10^3^/mmc)	208.5 (60–400)	221.5 (38–412)	0.157
INR (ratio)	1.13 (0.82–1.90)	1.19 (0.99–2.09)	0.097	INR (ratio)	1.04 (0.92–1.27)	1.08 (1.00–1.03)	0.001
Albumin (g/L)	3.60 (2.30–4.40)	3.60 (1.80–4.70)	0.631	Albumin (g/L)	4.00 (2.50–4.90)	3.99 (2.50–4.77)	0.429
Creatinin (mg/dL)	0.80 (0.44–3.00)	0.78 (0.45–1.52)	0.096	Creatinin (mg/dL)	0.82 (0.38–2.30)	0.80 (0.28–1.20)	0.505
ALT (IU/L)	40 (10–93)	40.5 (10–345)	0.445	ALT (IU/L)	31.5 (12–188)	33 (15–103)	0.307
AST (IU/L)	32 (11–80)	35 (6–226)	0.407	AST (IU/L)	31.5 (5–135)	32.5 (8–204)	0.675
Bilirubin (mg/dL)	1.09 (0.26–8.30)	1.01 (0.29–5.96)	0.802	Bilirubin (mg/dL)	0.69 (0.29–3.03)	0.66 (0.41–1.59)	0.834
Body mass index (kg/m^2^)	27.6 (22.2–35.6)	23.5 (20.1–28.2)	0.002	Body mass index (kg/m^2^)	28.1 (23.7.2–40.2)	23.8 (21.1–29.8)	<0.001
Total cholesterol (mg/dL)	166 (77–327)	148 (47–239)	0.255	Total cholesterol (mg/dL)	176.5 (69–267)	164.5 (117–237)	0.044
HDL cholesterol (mg/dL)	43 (9–97)	43.5 (8–132)	0.783	HDL cholesterol (mg/dL)	46 (16–78)	53.5 (24–124)	0.096
LDL cholesterol (mg/dL)	95 (17–231)	80.7 (25–163)	0.129	LDL cholesterol (mg/dL)	111.5 (21–186)	96.5 (32–156)	0.014
Tryglicerides (mg/dL)	92 (32–278)	75.5 (45–245)	0.080	Tryglicerides (mg/dL)	90 (19–275)	64.5 (32–148)	0.001
Child Pugh (*n*)				Spleen section area (cm^2^)	43 (22–83)	41.5 (21–97)	0.841
–A5	23 (53.5%)	23 (50.0%)	0.717	Diabetes (%)	5 (13.2%)	2 (6.2%)	0.442
–A6	8(18.5%)	14 (30.4%)		Metformin (%)	3 (7.9%)	1 (3.1%)	0.604
–B7	4 (9.3%)	3 (6.5%)		Insulin (%)	0	0	Not applicable
–B8	3 (7.0%)	3 (6.5%)		Statin (%)	8 (21.1%)	1 (3.1%)	0.033
–B9	3 (7.0%)	1 (2.2%)					
–C10	0	0					
–C11	2 (4.7%)	2 (4.3%)					
MELD (score)	10 (6–22)	9 (6–22)	0.392				
Spleen section area (cm^2^)	70 (30–180)	59.5 (23–150)	0.061				
Ascites	13 (30.2%)	9 (19.5%)	0.326				
Diabetes (%)	27 (62.8%)	9 (19.6%)	<0.001				
Metformin (%)	13 (30.2%)	1 (2.2%)	0.003				
Insulin (%)	12 (27.9%)	5 (10.9%)	0.059				
Statin (%)	10 (23.2%)	1 (2.2%)	<0.001				

INR: international normalized ratio; AST: aspartate aminotranferase; ALT: alanine aminotranferase; HDL: high-density lipoprotein; LDL: low-density lipoprotein.

**Table 4:** Univariate and multivariate binary logistic regression analysis to lysosomal acid lipase (LAL) in the subgroup of cirrhotic patients (performed using LAL below median as the dependent variable).

Univariate				Variable	Multivariate			
Exp (B)	95% CI for B		*p*		Exp (B)	95% CI for B		*p*
0.957	0.920	0.995	0.028	**Age (years)**	0.979	0.932	1.028	0.390
0.662	0.283	1.551	0.342	**Sex (F = 0, M = 1)**				
2.379	1.016	5.572	0.046	**Etiology (NAFLD = 1, HCV = 0)**	3.325	1.185	9.364	0.023
2.190	0.530	5.160	0.073	**HCC (NO = 0, YES = 1)**	1.560	0.187	1.675	0.300
1.977	0.851	4.594	0.113	**Oesophageal varices (NO = 0, YES = 1)**				
2.276	0.809	6.403	0.119	**Ascites (NO = 0, YES = 1)**				
1.000	0.999	1.000	0.007	**White blood cells (×10^3^/mm^3^)**	*			
1.000	0.999	1.000	0.064	**Neutrophils (×10^3^/mm^3^)**	1.000	0.999	1.000	0.439
0.999	0.998	1.000	0.005	**Lymphocytes (×10^3^/mm^3^)**	0.999	0.999	1.000	0.067
0.998	0.996	1.000	0.056	**Monocytes (×10^3^/mm^3^)**	1.000	0.997	1.003	0.759
0.985	0.977	0.994	0.001	**Platelets (×10^3^/mm^3^)**	0.989	0.980	0.999	0.028
69.620	2.986	1623.371	0.008	**INR (ratio)**	34.038	0.806	1436.912	0.065
0.911	0.431	1.925	0.806	**Albumin (g/dL)**				
0.517	0.171	1.559	0.242	**Creatinin (mg/dL)**				
0.991	0.979	1.003	0.127	**ALT (IU/L)**				
0.990	0.978	1.002	0.101	**AST (IU/L)**				
1.314	0.914	1.891	0.140	**Total bilirubin (mg/dL)**				
1.002	0.996	1.005	0.230	**Body mass index (kg/m^2^)**				
0.992	0.983	1.001	0.101	**Total cholesterol (mg/dL)**				
0.991	0.973	1.010	0.371	**HDL cholesterol (mg(dL)**				
0.992	0.981	1.004	0,199	**LDL cholesterol (mg/dL)**				
0.993	0.984	1.002	0.151	**Tryglycerides (mg/dL)**				
1.510	0.645	3.538	0.342	**Diabetes (NO = 0, YES = 1)**				
1.939	0.575	7.161	0.320	**Statin (NO = 0, YES = 1)**				
3.015	0.868	10.475	0.082	**Metformin (NO=0, YES=1)**	*			
0.889	0.308	2.562	0.827	**Insulin (NO = 0, YES = 1)**				
1.026	1.008	1.044	0.005	**Spleen sectional area (cm^2^)**	1.005	0.983	1.028	0.653
1.141	0.882	1.477	0.315	**Child-Pugh score**				
1.108	0.975	1.259	0.116	**MELD Score**				

* Excluded for multivariate analyses due to co-linearity. INR: international normalized ratio; AST: aspartate aminotranferase; ALT: alanine aminotranferase; HDL: high-density lipoprotein; LDL: low-density lipoprotein.

**Table 5:** Univariate and multivariate binary logistic regression analysis to lysosomal acid lipase (LAL) in the subgroup of non-cirrhotic patients (performed using LAL below median as the dependent variable).

Univariate				Variable	Multivariate			
Exp (B)	95% CI		*p*		Exp (B)	95% CI		*p*
0.986	0.957	1.015	0.346	**Age (years)**				
0.702	0.270	1.824	0.467	**Sex (F = 0, M = 1)**				
4.231	1.550	11.546	0.005	**Etiology (NAFLD = 1, HCV = 0)**	5.135	1.752	15.053	0.003
1.292	0.313	5.277	0.722	**HCC (NO = 0, YES = 1)**				
1.000	0.999	1.000	0.037	**White blood cells (×10^3^/mm^3^)**	*			
1.000	0.999	1.000	0.048	**Neutrophils (×10^3^/mm^3^)**	1.000	0.999	1.000	0.026
1.000	0.999	1.001	0.483	**Lymphocytes (×10^3^/mm^3^)**				
0.998	0.995	1.000	0.081	**Monocytes (×10^3^/mm^3^)**	0.998	0.996	1.001	0.182
0.995	0.988	1.002	0.166	**Platelets (×10^3^/mm^3^)**				
0.176	0.000	87.584	0.584	**INR (ratio)**				
0.574	0.207	1.596	0.288	**Albumin (g/dL)**				
0.264	0.032	2.173	0.216	**Creatinin (mg/dL)**				
0.982	0.960	1.005	0.125	**ALT (IU/L)**				
0.993	0.979	1.007	0.337	**AST (IU/L)**				
0.501	0.158	1.586	0.240	**Total bilirubin (mg/dL)**				
1.003	0.997	1.006	0.292	**Body mass index (kg/m^2^)**				
0.998	0.986	1.010	0.723	**Total cholesterol (mg/dL)**				
0.987	0.962	1.012	0.306	**HDL cholesterol (mg/dL)**				
0.998	0.984	1.012	0.784	**LDL cholesterol (mg/dL)**				
1.005	0.994	1.016	0.373	**Tryglycerides (mg/dL)**				
1.376	0.285	6.658	0.691	**Diabetes (NO = 0, YES = 1)**				
0.453	0.104	1.979	0.293	**Statin (NO = 0, YES = 1)**				
1.000	0.133	7.527	1.000	**Metformin (NO = 0, YES = 1)**				
1.014	0.978	1.051	0.453	**Spleen sectional area (cm^2^)**				

* Excluded for multivariate analyses due to co-linearity. INR: international normalized ratio; AST: aspartate aminotranferase; ALT: alanine aminotranferase; HDL: high-density lipoprotein; LDL: low-density lipoprotein.

## Abbreviations

LAL	lysosomal acid lipase
NAFLD	non-alcoholic fatty liver disease
HCV	hepatitis C virus
DBS	dried blood spots
WD	Wolman disease
CESD	cholesteryl ester storage disease
HCC	hepatocellular carcinoma
INR	international normalized ratio
ALT	alanine aminotransferase
AST	aspartate aminotransferase
MELD	model for end-stage liver disease
HBV	hepatitis B virus
DMSO	dimethyl sulfoxide
NCCLS	National Committee for Clinical Laboratory Standard
HDL	high density lipoproteins
